# Hygiene and the Health of Our Children

**Published:** 1919-12-27

**Authors:** 


### THE SCHOOL MEDICAL SERVICE.

### I. Hygiene and the Health of our Children.

The Annual Report for 1918 of the Chief Medical Office of the Board of Education is of the utmost interest in indicating the way in which the School Medical Service may render immense service to the State in the cause of national health and well-being. To bring forward more clearly the numerous points considered, we may, with advantage, deal in this article with those sections of the report bearing mainly on the incidence of disease in the schoolchild itself, and the methods adopted to meet it, and reserve for a later date discussion of the educative and preventive aspect of the work as it affects the normal child from infancy till maturity.

### The General Outlook.

In the first place, the scope of the school medical work has been widened by the Education Act of 1918, and the passing of the Ministry of Health Act of 1919 "has established the integral relationship of the School Medical Service to the other elements of the national scheme, which has for its object the improvement of the health of the people as a whole." The pioneer work of the medical service in the schools has been done. The organisation for diagnosis and treatment is there, and parents have already to a very large extent overcome their prejudices and have learnt to value the service highly. There is naturally room for improvement in certain directions which may be indicated later, but the foundations are laid, and it is time for the service to attempt constructive work on behalf of the nation as a whole. In the words of the report, "the State requires and expects a practical and national contribution from the School Medical Service now in being throughout the country."

Undoubtedly there exist immense possibilities far beyond the mere inspection, treatment, and following-up of individual cases in the schools, and it is the recognition of these possibilities which forms the inspiring feature of the report. "To the seeing eye and understanding mind," says Sir Geo. Newman, "the School Medical Service affords a most remarkable opportunity of establishing a high and enduring standard of national health. For here is the great recruitment; here is the nation in its childhood; here is the foundation of the future. Yet that is not all, for the School Medical Service, if rightly comprehended, provides the unique opportunity of preventive medicine. *It furnishes the occasion for the study of the beginnings of disease*—that great borderland of relationship between physiology and pathology, and between body and mind, wherein lie hidden so many of the secrets of the unknown; it furnishes the occasion for understanding the crippling effect of disease and its repair. . . ." The way now lies open to continuous and consecutive observation of symptoms through the schools for mothers, day nurseries, nursery schools, elementary and secondary schools, and then through the continuation classes and on into the factory or workshop. Normal and abnormal types can be watched and compared, the latter particularly through the special schools for defectives.

### Treatment and Following-up in the Schools.

It is natural that the war should have had a hindering effect upon the work in the schools, though in the actual mechanism of treatment there has been progress. The school clinics have gained in popularity, and in 1918 seventy more were established, making at the present time a total of 600 as against 7 in 1908. There is, of course, also treatment carried out at certain hospitals where arrangements are made by the local authorities. In certain districts temporary departures have been necessary from the Code of Regulations regarding medical inspection; this matter should now, however, automatically right itself. Also, a difficulty which has been felt during the war, especially in London, is the lack of sufficient numbers of voluntary workers to carry out the work of following-up. It appears to us that a grave problem is before the authorities in this matter, and one which should be faced squarely, certainly before the issue of the next report. It is of the utmost importance that this work should be done efficiently in order that the time and skill of the doctor should not be wasted, and educative opportunities should not be missed. A great responsibility rests upon care committees, and members of these committees must realise that their work is of the highest importance. Insistence must be made upon training. If properly-trained workers in sufficient numbers are not forthcoming, the present voluntary system, ideal though it be in theory, must be abandoned, and officials appointed who will be responsible for the "following-up" work by home visiting. The cost of the School Medical Service is increasing; the total sum spent by local authorities during the year ending March 1918 being £1,207,325—which is a large advance on the expenditure of the previous year. Much more, however, will have to be spent if the nation is to derive full benefit from the work and if money already spent is not to be wasted.

### Special Schools for Defective Children.

£500,000 of the above sum went towards the maintenance of special schools for blind, deaf, feeble-minded, epileptic, cripple, delicate, and tuberculous children. Many more such schools are needed. They are, as the report points out, run at great expense, and in many cases for the benefit of children who can never become useful citizens. Nevertheless these schools have an important function to fulfil not only in giving to the individual child the education best suited to its condition, mental or physical, but in discovering by means of observation and experiment new methods of education and treatment which may prove to be of infinite value. The work should be begun early and should not be hampered by lack of accommodation. Much also remains to be done in the fields of mental deficiency and of blindness.

**The School Medical Service—(*continued*).**

The Need for Open-Air Treatment.

We are in entire agreement with the report in the special emphasis laid on the need for more provision for open-air treatment, which is applicable both to the normal and abnormal child. Results have amply justified the existence of open-air schools in districts in which they have been established, and there is no need to dwell upon their importance here. Their curative and preventive value is surely unquestioned, yet only a score out of 318 local authorities have made use of the method, and "the life and education of an open-air school is only available for a few hundred children out of the hundreds of thousands who stand in need of it." As Sir George Newman says, "the slow growth in England of the open-air school methods seems scarcely creditable to our national good sense." It is to be hoped that all Local Education Authorities may be convinced ere long that from investment in this venture will their returns be largest and most certain. The King's Canadian Camp School for London children now established at Bushey is a recent step in the right direction. But among the many other ways in which open-air methods may be employed are classes in playgrounds, parks, etc., school journeys, holiday schools, and camps, and it is interesting to note in this connection the clause in the Education Act empowering Local Authorities to "make arrangements to supply or maintain or aid the supply or maintenance of holiday or school camps, especially for young persons attending continuation schools."

Tuberculosis in Childhood.

In the section headed thus, the report reminds us of Metchnikoff's saying in 1912 "that in his environment man encounters tubercle bacilli of very different degrees of virulence. It is during childhood that the contagion of these bacilli is established." The words of Dr. Batty Shaw are also quoted: "It is certain now that tuberculosis in childhood is universal." Statistics are given showing that, whatever may have been the special contributory causes in the form of influenza or lack of certain foodstuffs, the death-rate from tuberculosis of children between the ages of 5 and 15 has steadily risen since 1913. If then childhood is the susceptible period of life, the school medical officer has a large part to play in the campaign of prevention of tuberculosis. True, his responsibility is largely shared by the sanitary authorities, who have the control of the milk supply and general sanitary arrangements in their hands; the duties of the education authority, however, obviously include "for the diseased child early diagnosis and treatment, and for the susceptible child nutrition, a sanitary environment, and an open-air life." It is in connection with the susceptible child that the open-air school is of such high value. It is realised that the school doctor must himself be "an early diagnostician of tuberculosis," and work closely with the local tuberculosis officer. Mention is made in the report from Sheffield of the fact that there the school doctor assists once a week at the tuberculosis dispensary, and such an arrangement should prove most valuable. Particularly does this branch of health work call for comprehensive and concerted action, and throughout, the keynote of the report is a vision of live and workable schemes for treatment and education in the interests of national health. We have made selection of the most outstanding among the first, and must reserve until a later article the second, the educative, aspect, and one which deserves the country's closest attention.

